# Severe Hyponatremia in a 46-Year-Old Female With Pituitary Stalk Duplication and Primary Empty Sella Syndrome

**DOI:** 10.7759/cureus.43851

**Published:** 2023-08-21

**Authors:** Fatima Alkhyeli, Hessa Boharoon, Abdulla Almarzouqi

**Affiliations:** 1 Medical Education, Sheikh Khalifa Medical City, Abu Dhabi, ARE; 2 Endocrinology, Sheikh Khalifa Medical City, Abu Dhabi, ARE; 3 Internal Medicine, Sheikh Khalifa Medical City, Abu Dhabi, ARE

**Keywords:** hypogonadism, empty sella turcica, pituitary duplication, primary empty sella syndrome, hyponatremia

## Abstract

Pituitary duplication is a rare congenital malformation. It has been mainly reported in the pediatric and neonatal population, with few reported cases in the adult population. In this case report, we discuss the presentation of an adult female patient with pituitary stalk duplication and primary empty sella (PES).

A 46-year-old South Asian female presented with severe euvolemic hyponatremia. Initial investigation showed low serum osmolality, high urine osmolality, high urinary sodium concentration, and normal chest X-ray. On physical examination, the patient had underdeveloped secondary sexual characteristics. Laboratory tests showed low follicle-stimulating hormone, luteinizing hormone, and estradiol. Prolactin was moderately elevated, morning cortisol was low, adrenocorticotropic hormone (ACTH) was within the lower normal range, ACTH dynamic test was suboptimal, and insulin-like growth factor 1 was low. MRI showed empty sella with duplication of the pituitary stalk and third ventricle sagging.

Pituitary stalk duplication is a rare congenital malformation that can be associated with other craniofacial abnormalities. Here, we describe the occurrence of pituitary stalk duplication with PES. It is not known if the two conditions are associated with each other. However, we speculate that the duplication of the stalk might have altered the normal anatomy of the aperture in the sellar diaphragm creating a space for the third ventricle to sag or herniate, as well as compressing the pituitary gland.

## Introduction

Pituitary gland duplication is an exceedingly rare congenital malformation. Most cases reported in the literature described complete duplication of the pituitary gland. Partial duplication of the pituitary gland is less reported, and it involves duplication of the adenophysis and the pituitary stalk. In 2007, Kandpal et al. were the first to report two cases of partial duplication in adult patients [1]. Pituitary gland duplication is commonly associated with other craniofacial abnormalities such as duplication of the infundibulum, tubo-mamillary fusion, duplication of the basilar artery, agenesis/hypoplasia of the corpus callosum, hypertelorism, cleft palate, craniopharyngeal canal, oropharyngeal teratomas, and vertebral segmentation anomalies [2]. In this article, we report pituitary stalk duplication in a 46-year-old female with primary empty sella (PES). PES is defined as herniation of the subarachnoid space into the sella turcica due to sellar diaphragm deficiency with no history of previous pituitary disease. To our knowledge, the co-occurrence of these two conditions has not been reported previously.

## Case presentation

A 46-year-old South Asian female, with no known medical history, presented to our emergency department with nausea, vomiting, muscle cramps, dizziness, and fatigue worsening over the last week. On physical examination, blood pressure was 118/68 mmHg, heart rate was 80 beats per minute, and oxygen saturation was 99% on room air. The patient was dizzy, lethargic, and confused with dry skin and lips. Abdominal, chest, and neurological examinations were normal. Blood venous gas analysis revealed hyponatremia, measuring at 102 mmol/L. The patient was started on 3% NaCL and transferred to the intensive care unit (ICU). She was investigated for euvolemic hyponatremia. Initial investigation showed low serum osmolality and high urine osmolality and urinary sodium. Chest X-ray was normal.

On physical examination, it was noticed that the patient had underdeveloped secondary sexual characteristics, including the absence of breast development, no axillary hair, and scanty pubic hair. The patient revealed that she had scanty and irregular menstrual cycles from menarche till the age of 31. Since then, she had a complete cessation of her menstrual cycle. She was taken by her mother to a medical clinic during her teenage years for menstrual irregularities; however, no medical diagnosis was reached. She denied any medical conditions, previous surgeries, or similar history in her family.

Diagnostic assessment and management

Further laboratory tests were done (Table 1). Follicle-stimulating hormone (FSH), luteinizing hormone (LH), estradiol, insulin-like growth factor 1 (IGF-1), and morning cortisol levels were low. Adrenocorticotropic hormone (ACTH) was within the lower normal range. Prolactin was elevated, thyroid-stimulating hormone was normal, and the short Synacthen test (SST) was suboptimal (Table 2).

**Table 1:** Patient’s biochemical and hormonal profile with reference range. FSH = follicle-stimulating hormone; LH = luteinizing hormone; IGF-1 = insulin-like growth factor 1; ACTH = adrenocorticotropic hormone; TSH = thyroid-stimulating hormone

Laboratory test	Patient’s value	Normal range
Serum sodium	102 mmol/L	136–146 mmol/L
Serum osmolality	211 mOsmol/kg H_2_O	275–295 mOsmol/kg H_2_O
Urinary sodium	81 mmol/L	>40 mmol/L indicated increased urinary sodium concentration
Urinary osmolality	252 mOsm/L	50–1,200 mOsmol/kg H_2_O
FSH	0.8 milli mIU/mL	Premenopause: 4–30 mIU/mL; midcycle peak: 10–90 mIU/mL; postmenopause: 40–250 mIU/mL
LH	0.5 milli mIU/mL	Follicular phase: 5–30 mIU/mL; midcycle: 75–150 mIU/mL; postmenopause: 30–200 mIU/mL
Estradiol	<18 pmol/L	Proliferative phase: 220–918 pmol/L; luteal phase: 275–1,650 pmol/L; menopause: <40 pmol/L
Prolactin	1,760 μIU/mL	102–496 μIU/mL
IGF-1	3.26 nmol/L	10.3–28.6 nmol/L
Cortisol	123 nmol/L	140–690 nmol/L
ACTH	4.7 pmol/L	1.3–16.7 pmol/L
TSH	1.680 μU/mL	0.4–4.0 μU/mL

**Table 2:** Patient’s STT readings. ACTH = adrenocorticotropic hormone; SST = short Synecthan test

	Patient’s value	Interpretation
Cortisol at 0 minute	116 nmol/L	A cortisol of >420 nmol/L at 30 minutes post-Synacthen indicates an adequate adrenal response. Failure to meet the above indicates inadequate adrenal response due to primary adrenal disease or adrenal atrophy secondary to prolonged absence of ACTH stimulation
Cortisol at 30 minutes	383 nmol/L	
Cortisol at 60 minutes	424 nmol/L	

**Figure 1:**
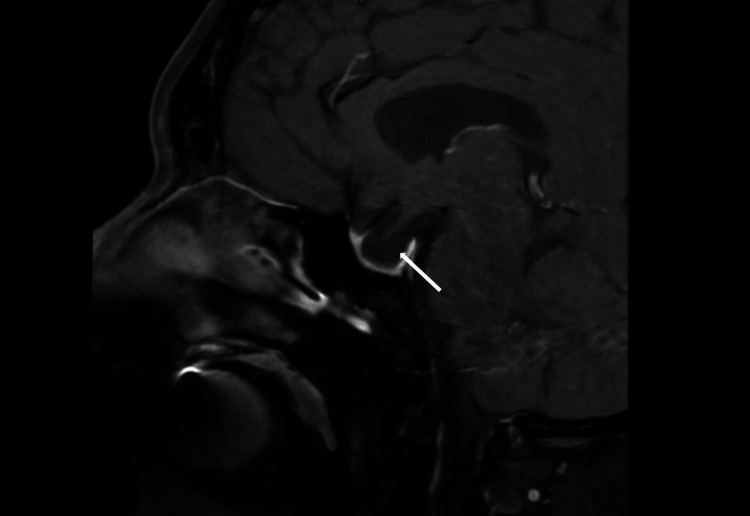
Sagittal T1-weighted MRI of the brain showing enlarged empty sella turcica (white arrow).

**Figure 2:**
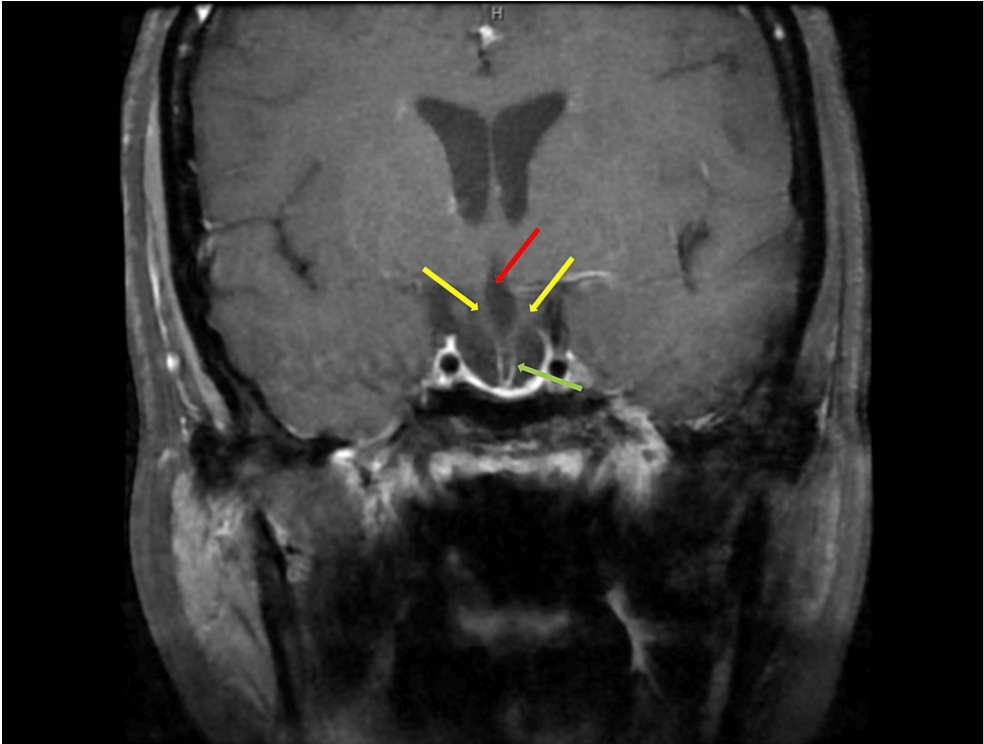
Coronal brain T1 imaging showing duplication of the pituitary stalk (yellow arrows) and sagging of the third ventricle floor (red arrow). Also, there is a ring-enhancing soft tissue compatible with the residual anterior pituitary gland (green arrow).

**Figure 3:**
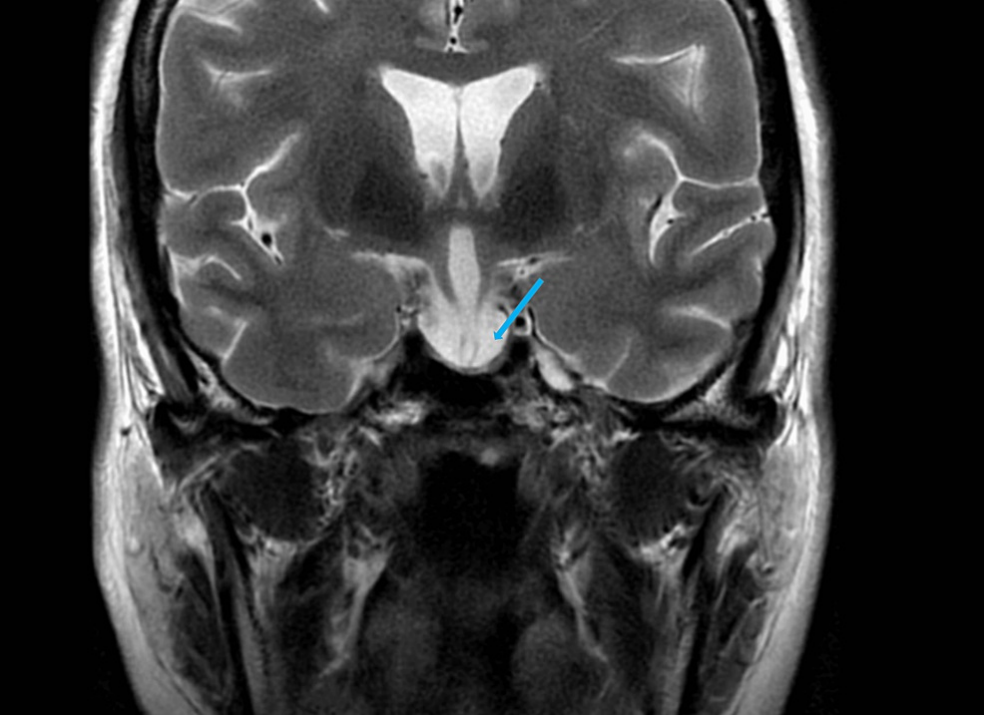
Coronal T2-weighted imaging showed the sella turcica filled with cerebrospinal fluid (blue arrow).

Sagittal T1-weighted MRI of the brain showed an enlarged empty sella turcica (Figure 1) and duplicated appearance of the pituitary stalk on coronal imaging with third ventricular herniation (Figure 2). In addition, there was ring-enhancing soft tissue on coronal T1-weighted imaging compatible with the residual anterior pituitary gland (Figure 2). T2-weighted imaging showed the filling of sella turcica with cerebrospinal fluid (CSF), which confirmed the radiological diagnosis of empty sella turcica (Figure 3). A Dual X-ray absorptiometry scan was done to measure bone density due to low estrogen levels, and the result was normal.

Management

Initially, the patient was managed in the ICU with hypertonic saline. Afterward, she was admitted to a high-dependency unit, and daily oral sodium tablets were administered. Due to her suboptimal response in the SST, the endocrine team suggested providing the patient with a steroid stress dose during stress or sickness. Once her sodium level stabilized at 130 mmol/L, the patient was discharged with oral sodium tablets along with a follow-up with the endocrinology and internal medicine clinic to further manage her hypogonadism and sodium levels. However, the patient failed to follow up with endocrinology due to a lack of medical insurance.

Diagnosis

Hyponatremia due to central adrenal insufficiency (CAI) and hypogonadotropic hypogonadism resulting from pituitary stalk duplication and PES.

## Discussion

Pituitary gland duplication was first described by Ahlfield in 1880 [3]. To date, only a few case reports have been reported mainly among neonatal and pediatric age groups [4-7]. It has also been reported in adults [8,9], however, only a few patients with pituitary gland duplication were able to survive beyond puberty due to the severity of associated craniofacial abnormalities. Pituitary gland duplication can be an incidental finding or can manifest in various clinical presentations as a result of abnormal pituitary hormone levels or due to associated craniovertebral anomalies.

In 1955, it was suggested by Morton that pituitary duplication and craniofacial abnormalities occur due to the splitting of cells of the prechordal plate and the anterior end of the notochordal process from the 15th to the 16th day of pregnancy [7]. However, the etiopathogenesis of notochord splitting remains unknown [10]. Several genes have been associated with pituitary development, including *RPX/HESX-1*, *PAX-6*, *SIX-1,3*, *Isl-1*, *PITX-1*, *PITX-2*, *PROP-1*, and many others [11]; however, their role in duplication of the pituitary gland is not well established.

Most cases have described complete duplication of the pituitary gland. Partial pituitary gland duplication is less reported and was first described in 2007 by Kandpal et al. [1]. Kandpal et al. reported two cases of partial gland duplication. Both cases were associated with other findings, including nasopharyngeal teratoma and macroadenoma. Other case reports of pituitary stalk duplication were associated with other abnormalities, including intrasellar dermoid cyst [12] and duplication of the hypothalamus [13], among others. Our patient had pituitary stalk duplication, with third ventricle sagging and PES. To our knowledge, this has not been described in the literature.

Empty sella turcica is defined as the herniation of the subarachnoid space into the sella turcica. It can be classified into primary or secondary empty sella. PES is caused by intracranial hypertension and sellar diaphragm insufficiency with no history of pituitary disease [14]. Secondary empty sella is more common than PES and occurs due to different factors, including pharmacological, radiological, and surgical treatment of the sellar region; infectious and inflammatory processes affecting the pituitary gland; and brain trauma [14]. Our patient had PES due to the absence of the latter in her medical history. The term primary empty sella syndrome is used when PES is associated with endocrine, neurologic, ophthalmologic, and psychiatric symptoms, such as in our patient.

Both empty sella and pituitary stalk duplication can lead to pituitary stalk compression which can, in turn, lead to hypopituitarism. Our patient had endocrinological manifestations including hypogonadism and CAI resulting from flattening of the pituitary gland and decreased hormonal secretion. In CAI, hypoosmolar hyponatremia occurs due to cortisol deficiency leading to increased corticotropin-releasing hormone (CRH) which is an antidiuretic hormone secretagogue [15]. Cortisol is a negative inhibitor of CRH and ACTH, and low levels of cortisol lead to loss of negative inhibition and increased levels of CRH [15]. The occurrence of severe hyponatremia in PES has been described in the literature [16-18].

We speculate that PES might be associated with pituitary stalk duplication. Normally, the pituitary stalk passes through an aperture in the sellar diaphragm to reach the hypophysis. Duplication of the pituitary stalk might alter the normal anatomy of the aperture creating a space for the third ventricle to sag or herniate. Herniation of the third ventricle compresses the pituitary gland and fills the sella turcica with CSF fluid which leads to pituitary hypoplasia. Other case reports of pituitary stalk duplication were also associated with third ventricle herniation, but empty sella turcica was not found on imaging [12,13]. In these two case reports, symptoms were reported early in childhood. This can be explained by associated abnormalities, such as anterior pituitary hypoplasia and intrasellar dermoid cyst. However, in the absence of other pituitary abnormalities, the formation of PES in pituitary stalk duplication might develop over time and manifest later in life, such as in our patient.

## Conclusions

Pituitary stalk duplication is a rare congenital malformation that can be associated with other craniofacial abnormalities. In this case report, we described the occurrence of pituitary stalk duplication with PES. The clinical manifestations in this patient occurred due to pituitary stalk compression leading to hypogonadism and CAI. CAI causes hyponatremia due to the loss of cortisol’s negative inhibition on CRH, which leads to increased antidiuretic hormone secretion and renal water absorption.

To our knowledge, the occurrence of pituitary stalk duplication with PES is undocumented in the literature. It is unknown if the conditions are associated. However, we speculate that duplication of the stalk might have altered the normal anatomy of the aperture creating a space for the third ventricle to sag or herniate and compress the pituitary gland. PES might have formed over time due to sellar diaphragm deficiency caused by stalk duplication, which can explain the late manifestations in our patient. We hope this case presentation proves to be helpful in understanding the clinical presentation of pituitary gland duplication in adults.
